# Rapid Capsular Antigen Immunoassay for Diagnosis of Inhalational Anthrax: Preclinical Studies and Evaluation in a Nonhuman Primate Model

**DOI:** 10.1128/mbio.00931-22

**Published:** 2022-05-12

**Authors:** Marcellene A. Gates-Hollingsworth, Cari B. Kolton, Alex R. Hoffmaster, Gabriel T. Meister, Addie E. Moore, Heather R. Green, Janice M. Pogoda, Segaran P. Pillai, Thomas R. Kozel

**Affiliations:** a Department of Microbiology and Immunology, University of Nevada, Reno School of Medicine, Reno, Nevada, USA; b Centers for Disease Control and Prevention, Atlanta, Georgia, USA; c Battelle Biomedical Research Center, Columbus, Ohio, USA; d Cipher Biostatistics & Reporting, Reno, Nevada, USA; e Office of the Commissioner, Food and Drug Administration, Silver Spring, Maryland, USA; University of California, Irvine

**Keywords:** anthrax, *Bacillus anthracis*, capsule, immunoassay, diagnosis, immunoassays

## Abstract

Inhalational anthrax is a fatal infectious disease. Rapid and effective treatment is critically dependent on early and accurate diagnosis. Blood culture followed by identification and confirmation may take days to provide clinically relevant information. In contrast, immunoassay for a shed antigen, the capsular polypeptide gamma-d-polyglutamate (γDPGA), can provide rapid results at the point of care. In this study, a lateral flow immunoassay for γDPGA was evaluated in a robust nonhuman primate model of inhalational anthrax. The results showed that the time to a positive result with the rapid test using either serum or blood as a clinical specimen was similar to the time after infection when a blood culture became positive. *In vitro* testing showed that the test was equally sensitive with cultures of the three major clades of Bacillus anthracis. Cultures from other *Bacillus* spp. that are known to produce γDPGA also produced positive results. The test was negative with human sera from 200 normal subjects and 45 subjects with culture-confirmed nonanthrax bacterial or fungal sepsis. Taken together, the results showed that immunoassay for γDPGA is an effective surrogate for blood culture in a relevant cynomolgus monkey model of inhalational anthrax. The test would be a valuable aid in early diagnosis of anthrax, which is critical for rapid intervention and a positive outcome. Use of the test could facilitate triage of patients with signs and symptoms of anthrax in a mass-exposure incident and in low-resource settings where laboratory resources are not readily available.

## INTRODUCTION

Bacillus anthracis is one of the most serious of the potential biothreat agents. Modeling studies suggest that aerosolized release of B. anthracis spores over a densely populated region could produce hundreds of thousands of illnesses and deaths with enormous economic impact (1, 2). The speed with which countermeasures can be effectively deployed, dispensed, and administered is critical to a successful response. However, a mass-casualty incident has the potential to rapidly overwhelm a local health care system and deplete essential medical countermeasures such as intravenous antibiotics and antitoxins if not effectively and selectively utilized (3, 4). The availability of a rapid diagnostic test that could differentiate between active inhalational anthrax infection on the one hand and the worried well, other forms of anthrax, or individuals who are not infected would focus available resources on those individuals most in need of treatment. An inexpensive rapid diagnostic test would also facilitate diagnosis in low-resource settings where anthrax may naturally occur.

The current gold standard for diagnosis of inhalational anthrax is the blood culture, which requires 24 h to a negative culture and 3 to 4 days to confirm positive cases (5). Blood culture has high sensitivity and specificity, but the time to acquire results from culture may not be conducive to initiate early treatment. Alternatives to blood culture include immunoassay for protective antigen (PA), assay for lethal factor (LF), and PCR for the *pagA* gene (6–9). Such tests would be used only if inhalational anthrax was suspected. Unfortunately, these tests vary in sensitivity, may require extensive sample processing, may take several hours to produce a result, may require complex instrumentation, or may require specific laboratory skills. A rapid, sensitive, and inexpensive point-of-care (POC) assay for presumptive diagnosis of inhalational anthrax complemented with clinical symptoms and epidemiological information would identify individuals most likely to benefit from early treatment that might not be possible with the currently existing tests.

Capsular antigens have been recognized as biomarkers for fungal and bacterial infections since the seminal report by Dochez and Avery that the capsular polysaccharide of Streptococcus pneumoniae can be detected via immunoassays using serum and urine from infected humans and rabbits (10). The presence of a secreted biomarker for anthrax was suggested by early studies by Ascoli and Valenti and Bail who found soluble antigen in body fluids of infected animals (cited in reference 10). B. anthracis resembles other encapsulated bacteria and fungi in the production of an antiphagocytic capsule; however, unlike other encapsulated microbes that produce polysaccharide capsules, B. anthracis produces a capsular polypeptide that is a homopolymer of d-glutamic acid residues that are linked via the γ-carboxyl (γDPGA) (11).

In previous studies, we demonstrated that detection of γDPGA in blood is an early indicator of infection in murine and rabbit models of inhalational anthrax (12, 13). γDPGA could be detected in serum at approximately the same time after challenge that a blood specimen subjected to culture (which required 24 h of incubation) became positive (13). These studies relied primarily on the enzyme-linked immunosorbent assay (ELISA) immunoassay platform and included use of lateral flow immunoassay (LFIA) for assessment of γDPGA in serum. The goal of the present study was to evaluate the extent to which a rapid, point-of-care immunoassay in LFIA format (Active Anthrax Detect [AAD]) could detect early-stage inhalational anthrax in a large-scale, robust, and physiologically relevant nonhuman primate (NHP) model of inhalational anthrax (14). In addition, we performed selected preclinical studies that assessed matrix effects, limits of detection, reactivity across clades of B. anthracis, reactivity with near neighbors of B. anthracis, reactivity with select bacteria which carry genes needed for synthesis of γDPGA, and reactivity with sera from healthy subjects and subjects with culture-confirmed sepsis due to nonanthrax bacteria and fungi.

## RESULTS

### Kinetics after challenge for (i) appearance of bacteremia, (ii) appearance of antigenemia, and (iii) alterations in hematology and blood chemistries.

**FIG 1:**
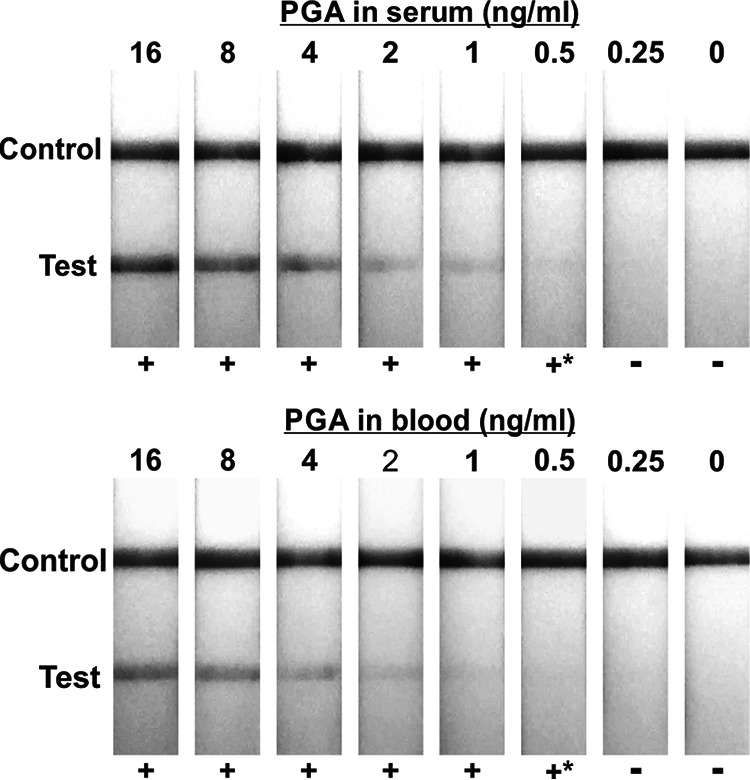
Limit of detection of the γDPGA LFIA tested with purified γDPGA diluted in pooled healthy human serum (top) and whole blood (bottom). Results from two blinded readers were considered positive if both observers read the strip as positive and considered negative if one or both read the strip as negative. The asterisk denotes a result that was weak but still deemed positive by both evaluators.

Ideally, diagnosis of anthrax in a bioterrorism exposure would utilize readily available clinical specimens such as whole blood or serum. As a consequence, an initial experiment assessed sensitivity of the AAD LFIA for detection of purified γDPGA in either serum or whole blood. γDPGA was spiked into and serially diluted in pooled human serum or whole blood, and reactivity was determined by visual inspection of the LFIA in strip format. The results (Fig. 1) showed readily visible results with γDPGA concentrations as low as 0.5 ng/mL.

With evidence that the LFIA can perform well using either serum or whole blood (Fig. 1), experiments were done to assess the kinetics for appearance of bacteremia and antigenemia after challenge, as well as alterations in several measures of hematology and clinical chemistry in a cynomolgus monkey model of inhalational anthrax. Five groups of 14 to 15 NHPs each were challenged by the pulmonary route with B. anthracis spores. Blood was collected at various times for assessment of (i) bacteremia via simple blood culture; (ii) numbers of bacteria (CFU per milliliter) via quantitative culture; (iii) presence of γDPGA in blood and serum; (iv) hematology for blood cell count, neutrophil counts, and lymphocyte counts; and (v) clinical chemistry to monitor aspartate aminotransferase, lactate dehydrogenase, and C-reactive protein. Presence of γDPGA was determined with the AAD LFIA using visual inspection as the readout. Evaluation of whole blood was done in real time at the point of sample collection. Evaluation of serum was done after separation of serum from blood. Blood was collected at 24 h after challenge and at 12-h intervals thereafter in experiments 1 and 2 and at 6-h intervals in experiments 3, 4, and 5. Time to positivity was coded as 0 (24 h), 1 (30 or 36 h), 2 (42 or 48 h), or 3 (>48 h).

**FIG 2:**
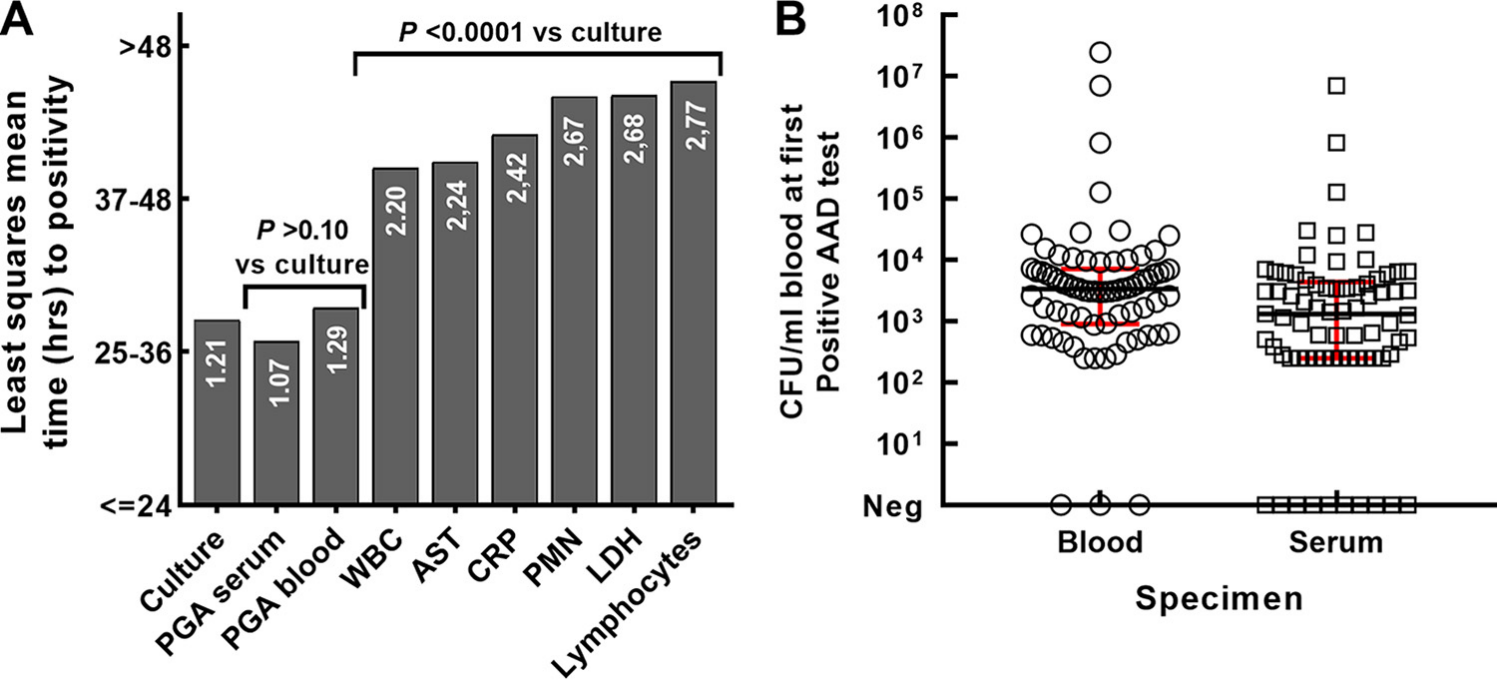
Performance of the AAD lateral flow immunoassay for γDPGA in an NHP model of inhalational anthrax. LFIAs were read visually 15 min after sample application. (A) Least-squares (LS) mean time interval to positivity by test. *P* values for comparisons to qualitative blood culture are shown above bars. LS means and *P* values are from a repeated-measures model with Dunnett’s adjustment for multiple comparisons. Time intervals for LS means were coded as follows: 0 = less than or equal to 24 h, 1 = 25 to 36 h, 2 = 37 to 48 h, and 3 = ≥48 h including censored. The time intervals are shown on each bar. Abbreviations: WBC, white blood cells; AST, aspartate aminotransferase; CRP, C-reactive protein; PMN, polymorphonuclear leukocytes; LDH, lactate dehydrogenase. (B) Distribution of quantitative culture for bacteremia at time of first positive AAD test when testing blood or serum. The horizontal line is the median; the length of box is the interquartile range. Each data point represents results from a single animal with a total of 73 animals in each column. Median number of CFU at the time of the first positive test for γDPGA in blood was 3,400; median number of CFU at the time of the first positive test for γDPGA in serum was 1,300. Negative culture results indicate either the absence of bacteremia or bacterial levels below the limit of detection at the time of the first positive AAD test.

Results from our testing showed that the blood culture and the AAD LFIA for γDPGA in blood and serum became positive early in the course of infection, approximately 24 to 36 h after challenge (Fig. 2A). There was no significant difference between the time of the first positive blood culture and a positive test for circulating γDPGA (*P* > 0.10 for serum or blood γDPGA versus blood culture). These results indicate that testing of serum or blood with the AAD LFIA is a rapid and effective surrogate for the blood culture. However, the AAD LFIA was able to provide a result in approximately 15 to 30 min after sample collection versus 24 h for blood culture.

Previous studies of the NHP model showed that a number of hematological and clinical chemistry parameters became abnormal in the course of inhalational anthrax (14). In agreement with this previous study, results from the present study found abnormal levels of aspartate aminotransferase, lactate dehydrogenase, C-reactive protein, white blood cell count, neutrophil counts, and lymphocyte counts late in the course of infection. All such abnormal values occurred 2 to 3 days after challenge, significantly later than either the first positive blood culture or the presence of detectable γDPGA in blood and serum (*P* < 0.0001) (Fig. 2A).

Quantitative blood culture was also done to determine the level of bacteremia (CFU per milliliter) at the time of the first positive test for antigenemia using serum or whole blood as a specimen (Fig. 2B). The median concentration of bacteria in blood at the time of the first positive test for γDPGA in whole blood was 3,370 CFU/mL (first quartile [Q1] = 940; Q3 = 7,130). The median concentration of bacteria at the time of the first positive test for γDPGA in serum was 1,330 CFU/mL (Q1 = 250; Q3 = 4,070). The difference was significant (*P* < 0.0001).

### Impact of sample matrix and immunoassay format on assay performance.

The AAD LFIA was developed in both dipstick and cassette formats. Both have advantages and disadvantages. As a consequence, the limit of detection for both formats was assessed using purified γDPGA that had been added to a variety of specimen matrices that might be considered for clinical use. Specimen matrices included serum; whole blood that had been treated with heparin, EDTA, or citrate; and capillary blood that had been treated with heparin or EDTA. Stock serial 2-fold dilutions of γDPGA in phosphate-buffered saline (PBS) prepared at 10× final desired concentration were used to spike each matrix; final γDPGA concentrations in each matrix ranged from 2 ng/mL to 0.135 ng/mL. Replicates were evaluated with each LFIA format at each antigen concentration, and the results were read by two independent blinded readers. The results showed a limit of detection of 0.5 to 1.0 ng/mL regardless of the assay format or specimen matrix (Table 1). Detailed results are provided in Tables S1 and S2 in the supplemental material.

**TABLE 1:** Impact of matrix and LFIA format on limit of detection

Matrix	Limit of detection (ng of γDPGA/mL)^*a*^	
	Strip	Cassette
Healthy human serum	1.0	1.0
Venous blood + EDTA	1.0	0.5
Venous blood + heparin	1.0	1.0
Venous blood + citrate	1.0	1.0
Capillary blood + EDTA	1.0	0.5
Capillary blood + heparin	1.0	1.0

a Limit of detection is the lowest concentration of γDPGA that produced a ≥95% positive average reading when two independent readers scored up to 30 replicate samples at each test concentration. All assays were produced by one operator and read in a blind manner by two other readers. Detailed results are provided in Tables S1 and S2.

10.1128/mbio.00931-22.1 TABLE S1 Effect of sample matrix on limit of detection for AAD strips using γDPGA that was spiked into the matrix. Download Table S1, DOCX file, 0.02 MB. Copyright © 2022 Gates-Hollingsworth et al. 2022 Gates-Hollingsworth et al. https://creativecommons.org/licenses/by/4.0/ This content is distributed under the terms of the Creative Commons Attribution 4.0 International license.

10.1128/mbio.00931-22.2 TABLE S2 Effect of sample matrix on limit of detection for the AAD cassette using PGA that was spiked into the matrix. Download Table S2, DOCX file, 0.02 MB. Copyright © 2022 Gates-Hollingsworth et al. 2022 Gates-Hollingsworth et al. https://creativecommons.org/licenses/by/4.0/ This content is distributed under the terms of the Creative Commons Attribution 4.0 International license.

### Reactivity of the AAD LFIA across clades of B. anthracis and with near neighbors.

Cultures were prepared from strains of B. anthracis from each of the three major clades of the bacterium (15). Serial dilutions were prepared using human serum as a matrix, and the reactivity was determined using the AAD LFIA. The results showed a high sensitivity for detection of B. anthracis from all three clades at bacterial concentrations as low as 100 to 1,000 CFU/mL (Table 2). Further studies were done to determine whether near neighbors of B. anthracis and other *Bacillus* spp. that do or do not produce capsule are reactive with the AAD LFIA (Table 3). Six species were evaluated. The presence of capsule for each strain was determined using immunofluorescence with capsular antiserum (16). Strains tested included two *Bacillus* spp. that do not demonstrate capsules and four *Bacillus* spp. or biovars that do produce glutamic acid capsules. For strains that do not produce capsule, the AAD LFIA results showed that broth cultures of Bacillus cereus ATCC 14579 were negative at 10^7^ CFU/mL. Bacillus thuringiensis ATCC 13367 was weakly positive at high concentrations (10^6^ CFU/mL) but negative at lower concentrations (10^3^ to 10^5^ CFU/mL). Although the B. thuringiensis strain showed cross-reactivity, this occurred at higher cell concentrations than for B. anthracis, and this species does not cause inhalation or septicemic disease in humans. Positive AAD LFIA results were observed with cultures of *Bacillus* spp. that do produce capsules: B. cereus biovar anthracis (CI), B. cereus biovar anthracis (CA), Bacillus megaterium, and Bacillus frigoritolerans.

**TABLE 2:** AAD LFIA results for strains from the three clades of B. anthracis at various concentrations

Approx CFU/mL	Result for strain:			
	New Hampshire (clade A)	Ames (clade A)	K2762 (clade B)	2002013094 (clade C)^*b*^
10^6^	Positive	ND^*a*^	Positive	ND
10^5^	Positive	Positive	Positive	ND
10^4^	Positive	Positive	Positive	ND
10^3^	Positive	Positive	Positive	Positive
10^2^	Negative	Positive	Weak positive	Weak positive
10^1^	Negative	Negative	Negative	Negative

a ND, not done.

b Strain 2002013094 (clade C) is a poor grower. Nevertheless, the limit of detection was similar to that of other strains.

**TABLE 3:** Reactivity of cultures of encapsulated and nonencapsulated *Bacillus* spp. with the AAD LFIA

Approx CFU/mL	B. cereus biovar anthracis (CI)^*a*^	B. cereus biovar anthracis (CA)^*a*^	B. megaterium 2008724125^*a*^	*B. frigoritolerans* 2008724126^*a*^	B. cereus ATCC 14579^*b*^	B. thuringiensis ATCC 13367^*b*^
10^7^	Positive	Positive	ND^*c*^	ND	Negative	ND
10^6^	Positive	Positive	ND	ND	ND	Weak positive
10^5^	Positive	Positive	Positive	Weak positive	ND	Negative
10^4^	Positive	Positive	Positive	Negative	ND	Negative
10^3^	Weak positive	Weak positive	Positive	Negative	ND	Negative
10^2^	Negative	Negative	Negative	Negative	ND	ND
10^1^	Negative	Negative	ND	ND	ND	ND

a *Bacillus* spp. or biovars that have capsules demonstrable by immunofluorescence using capsular antiserum.

b *Bacillus* spp. that lack capsules demonstrable by immunofluorescence using capsular antiserum.

c ND, not done.

The ability of cultures to produce a positive result in an immunoassay for γDPGA could be due to γDPGA that is either bound to the bacterial cells or shed or secreted, or both. Consequently, an experiment was done to assess the relative contributions of soluble versus cell-bound γDPGA to reactivity of bacterial cultures. Cultures of B. anthracis Ames were prepared, and serial dilutions were assessed using either filtered or unfiltered cells in a human serum matrix. The AAD LFIA results (Table 4) were the same for filtered and unfiltered cultures, indicating that shed γDPGA alone can account for the positive results.

**TABLE 4:** Reactivity of B. anthracis cultures with AAD can be caused by γDPGA that is shed into the culture fluid alone

Approx CFU/mL	B. anthracis Ames	
	Unfiltered culture	Culture filtrate
10^5^	Positive	Positive
10^4^	Positive	Positive
10^3^	Positive	Positive
10^2^	Positive	Positive
10^1^	Negative	Negative

### Failure to detect PGA with non-*Bacillus* bacteria with genes that code for γDPGA synthesis.

A bioinformatics analysis by Kocianova et al. found that Staphylococcus epidermidis, which contains a polyglutamate capsule consisting of approximately equal amounts of d- and l-glutamic acid, in contrast to B. anthracis, which contains only d-glutamic acid, contains the genes that code for γDPGA synthesis (17). Homologs of the *cap* locus are also found in a limited number of human pathogens, most notably Leptospira interrogans. Consequently, detection of γDPGA by two strains of S. epidermidis and three species of *Leptospira*, including five serovars of Leptospira interrogans, was assessed with broth cultures of each bacterium. In all cases, results with the AAD LFIA were negative (Table S3).

10.1128/mbio.00931-22.3 TABLE S3 Reactivity of AAD with cultures of bacteria that have genes that code for PGA synthesis. Download Table S3, DOCX file, 0.01 MB. Copyright © 2022 Gates-Hollingsworth et al. 2022 Gates-Hollingsworth et al. https://creativecommons.org/licenses/by/4.0/ This content is distributed under the terms of the Creative Commons Attribution 4.0 International license.

### Clinical specificity.

AAD in strip format was evaluated using sera from 200 normal subjects that were purchased from a commercial source. All tests were negative (data not shown). In addition, serum was obtained via a commercial source from 45 patients with a diagnosis of sepsis produced by 19 different bacteria or fungi that included *Bacillus* spp. (not *anthracis*), Candida tropicalis, Escherichia coli, Staphylococcus aureus, S. epidermidis, and Streptococcus pneumoniae (full list in Table S4). All tested negative in the AAD LFIA.

10.1128/mbio.00931-22.4 TABLE S4 Evaluation of AAD with serum from patients with sepsis. Download Table S4, DOCX file, 0.01 MB. Copyright © 2022 Gates-Hollingsworth et al. 2022 Gates-Hollingsworth et al. https://creativecommons.org/licenses/by/4.0/ This content is distributed under the terms of the Creative Commons Attribution 4.0 International license.

## DISCUSSION

Our results indicate that testing for γDPGA in blood is an effective surrogate for blood culture for early diagnosis of inhalational anthrax in a relevant animal model. The time to a positive result using either serum or whole blood as a clinical specimen was similar to the time after infection when a blood culture became positive (*P* > 0.10 for serum or whole blood versus culture). Moreover, the concentration of bacteria in blood at the time of the first positive AAD result (1,300 to 3,400 CFU/mL) (Fig. 2) was similar to the concentration of bacteria in culture that produced a positive result (approximately 1,000 CFU/mL) (Table 2). However, in contrast to the blood culture, which can take days to a confirmed positive result, the AAD test provided results in 15 min without the need for a sophisticated microbiology laboratory. Importantly, AAD provided rapid results when whole blood was collected from animals and the test was performed directly at the site of collection with appropriate proper personal protective equipment and a medical/hazardous waste management system.

The AAD test meets the ASSURED criteria that describe a diagnostic that is well suited for use in both developed and resource-limited environments (18). Lateral flow tests are typically low-complexity tests and highly affordable (18). Sensitivity was similar to that of the blood culture in the NHP model (Fig. 1). Although further testing is needed, specificity testing showed no reactivity with normal sera or sera from patients with sepsis caused by a variety of bacteria (see Table S4 in the supplemental material). AAD is user friendly, requiring few steps, and has a platform technology that is familiar to most potential users. Results are rapid (15 min) and robust, perform well with serum or whole blood, and are compatible with available anticoagulants. In addition, identical results were observed when the test was performed in a strip format and in a strip that was enclosed in a cassette. The test requires no equipment and is read visually. Deliverability is yet to be determined but would be similar to that of other rapid tests.

Although inhalational anthrax is one of the most severe of the biothreats, the actual frequency of inhalational anthrax in the absence of a deliberate release is quite rare. As a consequence, studies of the pathophysiology, diagnosis, and treatment of inhalational anthrax have had to rely on animal model studies. The FDA Animal Rule was developed for evaluation of new drugs when human efficacy studies are not ethical and field trials after an accidental or deliberate exposure are not feasible (FDA Animal Rule; 21 CFR 314.600 for drugs and 21 CFR 601.90 for biological products). The cynomolgus macaque anthrax inhalational model used in this study is well described and has been proposed for testing potential anthrax therapeutics in accordance with the FDA Animal Rule (14). The present study is a robust, large-scale (73 animals) application of this model to evaluate the AAD diagnostic LFIA.

We previously reported that γDPGA in serum is a biomarker for anthrax infection in murine, rabbit, and nonhuman primate models of inhalational anthrax (12, 13, 19). These earlier studies used serum or plasma as a specimen and an ELISA for detection of γDPGA. In the present study, we demonstrate that a point-of-care lateral flow assay can be used in real time for diagnosis of early-stage infection. The test can be performed with whole blood at the location where blood is collected without the need for a laboratory. We also provide results from *in vitro* studies for specificity, inclusivity, and sensitivity (CFU per milliliter). Finally, the present study used a large number of animals to provide confidence in our conclusions.

Inclusivity studies done *in vitro* found similar positive AAD results with representative strains of the three major clades of B. anthracis. Positive results were also found with cultures of other *Bacillus* spp. that are known to produce capsules demonstrable by immunofluorescence, including B. cereus biovar anthracis, B. megaterium, and B. frigoritolerans. Positive results with isolates of B. cereus biovar anthracis from Côte d’Ivoire (CI) and Cameroon (CA) are important because B. cereus biovar anthracis produces an anthrax-like disease in wild and domestic mammals in tropical Africa and is a Tier 1 select agent. No reactivity was observed with a culture of B. cereus which does not produce an observable capsule *in vitro*, and very weak reactivity was found with B. thuringiensis. B. thuringiensis is an insect pathogen that typically does not produce a γDPGA capsule; however, a γDPGA capsule has been noted in at least one strain (20).

One potential concern with detection of γDPGA as a surrogate for the blood culture is the possibility of false-positive reactions. As noted above, production of γDPGA is not limited to B. anthracis. For example, Bacillus licheniformis produces a capsular polypeptide that is comprised of γPGA in the d- and l-isoforms (21, 22). In addition, some bacteria contain genes that code for γDPGA synthesis (17). However, available evidence suggests that the potential for false-positive results from non-anthrax-producing bacteria may be minimal. First, most non-*anthracis* species of *Bacillus* such as B. megaterium and *B. frigoritolerans* do not cause clinical disease that might be confused as anthrax. Pseudoinfection due to contamination of specimens by *Bacillus* spp. is far more likely than a true infection (23). Second, positive results were not observed with cultures of S. epidermidis or *Leptospira* spp. (Table S3), bacteria that have genes that code for γDPGA production (17). Finally, no false-positive results were found with sera from 200 healthy subjects and 45 patients with a diagnosis of microbial sepsis that included S. epidermidis and *Bacillus* spp. (not *anthracis*).

There is a caveat to use of blood culture for diagnosis of the earliest phases of bacteremia in the NHP model. Serial sampling of blood in the NHP model is limited to relatively small blood volumes at each time point. In contrast, Clinical and Laboratory Standards Institute (CLSI) guidelines for blood culture for human infection recommend collection of 4 to 6 10-mL bottles for evaluation of bacteremia (24). As a consequence, blood culture in the NHP model may miss very low levels of bacteremia compared to culture using CLSI guidelines due to differences in sample volumes. However, blood culture takes as many as 24 h for positive growth and 3 to 4 days to confirm identification of B. anthracis and is not suited for rapid POC use. This difference in specimen volumes between the NHP model and CLSI guidelines suggests that testing for γDPGA may not be well suited for screening of asymptomatic individuals in the earliest stages of infection. However, the AAD test would likely be a rapid and effective surrogate for blood culture in the symptomatic patient.

There are multiple applications of the AAD test for diagnosis of anthrax. First, the test could be used in a mass-exposure incident for triage of patients with signs and symptoms of inhalational anthrax. Second, the test could identify patients who would best benefit from use of countermeasures that are in short supply. Third, the test could be of value in cases of inhalational anthrax where blood cultures are negative due to receiving prior antibiotics. Fourth, a test that meets the ASSURED criteria for a diagnostic could be of great value for diagnosis of anthrax in resource-limited settings. Finally, anthrax is enzootic in many regions (25). Humans may become infected by exposure to or through consumption of meat from infected animals. AAD could rapidly identify infected animal carcasses (26).

In summary, AAD is a rapid, sensitive, and user-friendly tool for diagnosis of anthrax. The test works by detection of capsular antigen that is shed into blood during infection. Unlike the blood culture, the lateral flow immunoassay can provide results in 15 min at the point of sample collection without the need for a microbiology laboratory. The test has the potential to rapidly triage patients who have been exposed to the microbe and have symptoms suggestive of anthrax. Such use would enhance patient care and identify patients most in need of medical countermeasures such as toxin-neutralizing antibodies that might be in short supply. Finally, γDPGA is an attractive biomarker for infection because the γDPGA is an essential virulence factor that cannot be engineered to avoid surveillance (27).

## MATERIALS AND METHODS

### Bacterial cultures.

B. anthracis Pasteur was used for isolation of γDPGA. The Pasteur strain is maintained by the Nevada State Public Health Laboratory (Reno, NV) and was originally obtained from the Centers for Disease Control and Prevention (CDC). All other strains used are maintained in the CDC’s collection and include four B. anthracis strains (Ames, New Hampshire, K2762, and 2002013094), B. cereus ATCC 14579, B. thuringiensis ATCC 13367, B. cereus G9241, two B. cereus biovar anthracis strains (CA and CI), B. megaterium 2008724125, *B. frigoritolerans* 2008724126, Staphylococcus epidermidis (ATCC 35984 and ATCC 12228), and seven *Leptospira* serovars (L. interrogans serovar Australis Ballico, Leptospira borgpetersenii serovar Ballum Mus 127, L. interrogans serovar Bataviae Van Tienen, L. interrogans serovar Canicola Ruebush, L. interrogans serovar Icterohaemorrhagiae RGA, Leptospira santarosai serovar Georgia LT 117, and L. interrogans serovar Pomona Pomona).

### NHP model of inhalational anthrax.

All animal procedures were approved by Battelle’s Institutional Animal Care and Use Committee, and all exposures and assays were performed in a biosafety level 3 laboratory registered with the Centers for Disease Control and Prevention.

Cynomolgus macaques (Macaca fascicularis) were aerosol challenged with a targeted dose of 200 50% lethal doses (LD_50_) of B. anthracis Ames strain spores (1.24 × 10^7^ spores total [28]). Details regarding the inhalational model have been described previously (14). Venous blood was collected beginning 24 h postchallenge from NHPs included in an NIAID-funded study to evaluate potential medical countermeasures against anthrax. Residual blood samples that were analyzed by LFIA represented a subset of the animals in the original study. The animals analyzed by LFIA were divided into five groups of 14 to 15 NHPs for a total of 73 animals. In experiments 1 and 2, blood was collected for analysis at 12-h intervals. In experiments 3, 4, and 5, blood was collected at 6-h intervals. Routine qualitative blood culture assay was performed by three-phase streaking of 30 to 40 μL of blood onto a blood agar plate. For quantitative bacteremia culture, undiluted blood as well as serial dilutions of 1:10 was prepared and spread plate in triplicate over Trypticase soy agar (TSA) plates (100 μL/plate). Growth of colonies with morphology consistent with B. anthracis was indicated as positive or negative for standard blood culture. Colonies present on plates prepared for quantitative bacteremia were counted and reported as CFU per milliliter. The lower limit of quantitation was considered ≤250 CFU/mL. Standard clinical pathology (clinical chemistry and hematology) parameters were also assessed at each time point. Parameters of interest included white blood cells, polymorphonuclear leukocytes, lymphocytes, aspartate aminotransferase, C-reactive protein, and lactate dehydrogenase.

### Statistics.

For time-to-positivity analysis, collection times were coded as 0 = 24 h, 1 = 30 or 36 h, 2 = 42 or 48 h, and 3 = >48 h (including censored). For comparisons to culture on time to positivity, a repeated-measures analysis was done using a marginal linear model with unstructured covariance, with coded collection time as the dependent variable and test (γDPGA serum, γDPGA blood, etc.) as the fixed effect. Dunnett’s test was used to control for multiplicity. For paired comparisons of median bacteremia level at first positive test in blood versus serum, a signed-rank test was used. Hypothesis tests were two-sided with 0.05 significance levels. Statistical analyses were done using SAS v9.4 (SAS Institute Inc., Cary, NC).

### γDPGA production by cultures of B. anthracis and growth conditions of other bacteria.

γDPGA was isolated from B. anthracis Pasteur as previously described (12, 29) and used for spiking experiments in the different matrices. For testing the AAD LFIA, spore-forming bacteria were streaked for isolation onto Trypticase soy agar plates containing 5% sheep blood (SBA) from frozen stocks stored in 25% glycerol. Multiple single colonies were used to inoculate capsule-production medium (heart infusion broth containing horse serum and 8% sodium bicarbonate) and incubated for approximately 18 to 20 h at 37°C in 5% CO_2_. Dilutions (1:10) up to 10^7^ were performed in pooled human sera and tested using the AAD LFIA. Bacterial concentrations (CFU per milliliter) were calculated by plating 100 μL of dilutions in triplicate onto SBA and incubating overnight at 37°C. Dilution plates with countable colonies (30 to 300) were averaged per dilution.

S. epidermidis strains were streaked for isolation and inoculated into capsule-producing medium as described above, except that tryptic soy broth with and without 1 M sodium chloride was used, and incubation was with shaking at 37°C for 24 h in ambient atmosphere. *Leptospira* spp. were grown in liquid EMJH medium at 30°C for 4 days, and turbidity was adjusted to a 0.5 McFarland standard.

### Lateral flow immunoassay for γDPGA.

The AAD LFIA was provided by InBios, International. AAD is an antigen-capture immunoassay that is constructed from monoclonal antibody (MAb) 8B10, which is reactive with γDPGA. Details regarding construction and use of the AAD LFIA have been described previously (13, 26). Lateral flow assays were constructed in both dipstick and cassette formats. The LFIA testing of NHP samples was done solely with assays in cassette format. Assays were read visually 15 min after application of the clinical sample.

### Human specimens.

Serum, whole blood, and capillary blood were purchased from a commercial vendor (BioIVT, Durham, NC). Pooled normal human serum and sera from 200 individuals were also purchased from BioVT. Additionally, 45 serum samples from culture-confirmed sepsis patients were acquired from BioIVT.
