# Supplementary material for: Rapid Capsular Antigen Immunoassay for Diagnosis of Inhalational Anthrax: Preclinical Studies and Evaluation in a Nonhuman Primate Model
Source: mBio. 2022 May 12;13(3):e00931-22. doi: 10.1128/mbio.00931-22 (PMC9239138; doi:10.1128/mbio.00931-22)
Supplement: TABLE S1 [file mbio.00931-22-s0001.docx]

| **Supplementary Table 1. Effect of sample matrix on limit of detection for AAD strips using γDPGA that was spiked into the matrix** | | | | | | | |
| --- | --- | --- | --- | --- | --- | --- | --- |
| **Matrix^a^** | **PGA^b^ concentration (ng/ml)** | **Operator #1** | | **Operator #2** | | **Average of 2 operators** | |
|  |  | **Positive/**  **Total** | **% Positive** | **Positive/**  **Total** | **% Positive** | **Positive/**  **Total** | **% Positive** |
| Human serum 1 | 0 | 0/30 | 0 | 0/30 | 0 | 0/30 | 0 |
|  | 0.12 | 0/30 | 0 | 0/30 | 0 | 0/30 | 0 |
|  | 0.25 | 2/30 | 6.7 | 0/30 | 0 | 1/30 | 3.4 |
|  | 0.50 | 30/30 | 100 | 25/30 | 83 | 27.5/30 | 92 |
|  | 1.0 | 30/30 | 100 | 29/30 | 97 | 29.5/30 | 99 |
|  | 2.0 | 30/30 | 100 | 30/30 | 100 | 30/30 | 100 |
| Human serum 2^c^ | 0 | 0/12 | 0 | 1/12 | 8.3 | 0.5/12 | 4.2 |
|  | 0.12 | 4/12 | 33 | 0/12 | 0 | 2/12 | 17 |
|  | 0.25 | 11/12 | 92 | 6/12 | 50 | 8.5/12 | 71 |
|  | 0.50 | 12/12 | 100 | 12/12 | 100 | 12/12 | 100 |
|  | 1.0 | 12/12 | 100 | 12/12 | 100 | 12/12 | 100 |
|  | 2.0 | 12/12 | 100 | 12/12 | 100 | 12/12 | 100 |
| Venous blood with EDTA | 0 | 1/30 | 3.3 | 1/30 | 3.3 | 1/30 | 3.3 |
|  | 0.12 | 1/30 | 3.3 | 0/30 | 0 | 0.5/30 | 1.7 |
|  | 0.25 | 5/30 | 17 | 5/30 | 17 | 5/30 | 17 |
|  | 0.50 | 30/30 | 100 | 26/30 | 87 | 28/30 | 94 |
|  | 1.0 | 30/30 | 100 | 28/30 | 93 | 29/30 | 97 |
|  | 2.0 | 30/30 | 100 | 30/30 | 100 | 30/30 | 100 |
| Venous blood with heparin | 0 | 2/30 | 6.7 | 0/30 | 0 | 1/30 | 3.4 |
|  | 0.12 | 0/30 | 0 | 0/30 | 0 | 0/30 | 0 |
|  | 0.25 | 1/30 | 3.3 | 1/30 | 3.3 | 1/30 | 3.3 |
|  | 0.50 | 30/30 | 100 | 26/30 | 87 | 28/30 | 94 |
|  | 1.0 | 30/30 | 100 | 30/30 | 100 | 30/30 | 100 |
|  | 2.0 | 30/30 | 100 | 30/30 | 100 | 30/30 | 100 |
| Venous blood with citrate | 0 | 1/30 | 3.3 | 1/30 | 3.3 | 1/30 | 3.3 |
|  | 0.12 | 1/30 | 3.3 | 0/30 | 0 | 0.5/30 | 1.7 |
|  | 0.25 | 2/30 | 6.7 | 0/30 | 0 | 1/30 | 3.4 |
|  | 0.50 | 30/30 | 100 | 26/30 | 87 | 28/30 | 94 |
|  | 1.0 | 30/30 | 100 | 30/30 | 100 | 30/30 | 100 |
|  | 2.0 | 30/30 | 100 | 30/30 | 100 | 30/30 | 100 |
| Capillary blood with EDTA | 0 | 2/30 | 6.7 | 1/30 | 3.3 | 1.5/30 | 5 |
|  | 0.12 | 0/30 | 0 | 0/30 | 0 | 0/30 | 0 |
|  | 0.25 | 2/30 | 6.7 | 1/30 | 3.3 | 1.5/30 | 5 |
|  | 0.50 | 27/30 | 90 | 24/30 | 80 | 25.5/30 | 85 |
|  | 1.0 | 30/30 | 100 | 30/30 | 100 | 30/30 | 100 |
|  | 2.0 | 30/30 | 100 | 30/30 | 100 | 30/30 | 100 |
| Capillary blood with heparin | 0 | 1/30 | 3.3 | 0/30 | 0 | 0.5/30 | 1.7 |
|  | 0.12 | 0/30 | 0 | 0/30 | 0 | 0/30 | 0 |
|  | 0.25 | 3/30 | 10 | 0/30 | 0 | 1.5/30 | 5 |
|  | 0.50 | 29/30 | 97 | 20/30 | 67 | 24.5/30 | 82 |
|  | 1.0 | 30/30 | 100 | 30/30 | 100 | 30/30 | 100 |
|  | 2.0 | 30/30 | 100 | 30/30 | 100 | 30/30 | 100 |
| ^a^ Serum, whole blood with different anticoagulants and capillary blood were purchased from Bioreclamation.  ^b^ γDPGA was spiked into the various matrices at the indicated concentrations. Unless otherwise indicated, 30 replicates at each antigen concentration.  ^c^ The number of available strips was limited for evaluation of the second lot of serum; only 12 strips were tested per concentration | | | | | | | |
