# Supplementary material for: Rapid Capsular Antigen Immunoassay for Diagnosis of Inhalational Anthrax: Preclinical Studies and Evaluation in a Nonhuman Primate Model
Source: mBio. 2022 May 12;13(3):e00931-22. doi: 10.1128/mbio.00931-22 (PMC9239138; doi:10.1128/mbio.00931-22)
Supplement: TABLE S2 [file mbio.00931-22-s0002.docx]

| **Supplementary Table 2. Effect of sample matrix on limit of detection for the AAD cassette using PGA that was spiked into the matrix.** | | | | | | | |
| --- | --- | --- | --- | --- | --- | --- | --- |
| **Matrix^a^** | **PGA^b^ concentration (ng/ml)** | **Operator #1** | | **Operator #2** | | **Average of 2 operators** | |
|  |  | **Positive/**  **Total** | **% Positive** | **Positive/**  **Total** | **% Positive** | **Positive/**  **Total** | **% Positive** |
| Human serum 1 | 0 | 0/30 | 0 | 0/30 | 0 | 0/30 | 0 |
|  | 0.12 | 0/30 | 0 | 0/30 | 0 | 0/30 | 0 |
|  | 0.25 | 1/30 | 3.3 | 1/30 | 3.3 | 1/30 | 3.3 |
|  | 0.50 | 29/30 | 97 | 22/30 | 73 | 25.5/30 | 85 |
|  | 1.0 | 30/30 | 100 | 30/30 | 100 | 30/30 | 100 |
|  | 2.0 | 30/30 | 100 | 30/30 | 100 | 30/30 | 100 |
| Human serum 2 | 0 | 0/30 | 0 | 0/30 | 0 | 0/30 | 0 |
|  | 0.12 | 1/30 | 3.3 | 0/30 | 0 | 0.5/30 | 1.7 |
|  | 0.25 | 23/30 | 77 | 5/30 | 17 | 14/30 | 47 |
|  | 0.50 | 30/30 | 100 | 30/30 | 100 | 30/30 | 100 |
|  | 1.0 | 30/30 | 100 | 30/30 | 100 | 30/30 | 100 |
|  | 2.0 | 30/30 | 100 | 30/30 | 100 | 30/30 | 100 |
| Venous blood with EDTA | 0 | 0/30 | 0 | 1/30 | 3.3 | 0.5/30 | 1.7 |
|  | 0.12 | 0/30 | 0 | 0/30 | 0 | 0/30 | 0 |
|  | 0.25 | 2/30 | 6.7 | 0/30 | 0 | 1/30 | 3.4 |
|  | 0.50 | 28/30 | 93 | 29/30 | 97 | 28.5/30 | 95 |
|  | 1.0 | 29/30 | 97 | 29/30 | 97 | 29/30 | 97 |
|  | 2.0 | 30/30 | 100 | 30/30 | 100 | 30/30 | 100 |
| Venous blood with heparin | 0 | 0/30 | 0 | 0/30 | 0 | 0/30 | 0 |
|  | 0.12 | 0/30 | 0 | 0/30 | 0 | 0/30 | 0 |
|  | 0.25 | 0/30 | 0 | 1/30 | 3.3 | 0.5/30 | 1.7 |
|  | 0.50 | 25/30 | 83 | 21/30 | 70 | 23/30 | 77 |
|  | 1.0 | 30/30 | 100 | 30/30 | 100 | 30/30 | 100 |
|  | 2.0 | 30/30 | 100 | 30/30 | 100 | 30/30 | 100 |
| Venous blood with citrate | 0 | 0/30 | 0 | 0/30 | 0 | 0/30 | 0 |
|  | 0.12 | 0/30 | 0 | 0/30 | 0 | 0/30 | 0 |
|  | 0.25 | 1/30 | 3.3 | 0/30 | 0 | 0.5/30 | 1.7 |
|  | 0.50 | 30/30 | 100 | 24/30 | 80 | 27/30 | 90 |
|  | 1.0 | 30/30 | 100 | 30/30 | 100 | 30/30 | 100 |
|  | 2.0 | 30/30 | 100 | 30/30 | 100 | 30/30 | 100 |
| Capillary blood with EDTA | 0 | 0/30 | 0 | 0/30 | 0 | 0/30 | 0 |
|  | 0.12 | 0/30 | 0 | 0/30 | 0 | 0/30 | 0 |
|  | 0.25 | 0/30 | 0 | 0/30 | 0 | 0/30 | 0 |
|  | 0.50 | 30/30 | 100 | 30/30 | 100 | 30/30 | 100 |
|  | 1.0 | 30/30 | 100 | 30/30 | 100 | 30/30 | 100 |
|  | 2.0 | 29/30 | 97 | 29/30 | 97 | 29/30 | 97 |
| Capillary blood with heparin | 0 | 0/30 | 0 | 0/30 | 0 | 0/30 | 0 |
|  | 0.12 | 0/30 | 0 | 0/30 | 0 | 0/30 | 0 |
|  | 0.25 | 0/30 | 0 | 0/30 | 0 | 0/30 | 0 |
|  | 0.50 | 29/30 | 97 | 24/30 | 80 | 26.5/30 | 89 |
|  | 1.0 | 30/30 | 100 | 29/30 | 97 | 29.5/30 | 99 |
|  | 2.0 | 30/30 | 100 | 30/30 | 100 | 30/30 | 100 |
| ^a^ Serum, whole blood with different anticoagulants and capillary blood were purchased from Bioreclamation.  ^b^ PGA was spiked into the various matrices at the indicated concentrations. Thirty replicate tests were evaluated at each antigen concentration. | | | | | | | |
