# Supplementary material for: Rapid Capsular Antigen Immunoassay for Diagnosis of Inhalational Anthrax: Preclinical Studies and Evaluation in a Nonhuman Primate Model
Source: mBio. 2022 May 12;13(3):e00931-22. doi: 10.1128/mbio.00931-22 (PMC9239138; doi:10.1128/mbio.00931-22)
Supplement: TABLE S3 [file mbio.00931-22-s0003.docx]

| **Supplemental Table 3. Reactivity of AAD with cultures of bacteria that have genes that code for PGA synthesis** | |
| --- | --- |
| **Bacteria** | **AAD reactivity** |
| *L. borgpetersenii* serovar Ballum Mus 127 | Negative |
| *L. interrogans* serovar Australis Ballico | Negative |
| *L. interrogans* serovar Bataviae Van Tienen | Negative |
| *L. interrogans* serovar Canicola Ruebush | Negative |
| *L. interrogans* serovar Icterohaemorrhagiae RGA | Negative |
| *L. interrogans* serovar Pomona Pomona | Negative |
| *L. santarosai* serovar Georgia LT 117 | Negative |
| *Staphylococcus epidermidis* ATCC 35984^a^ | Negative |
| *Staphylococcus epidermidis* ATCC 12228^a^ | Negative |
| ^a^ *S. epidermis* strains were cultured in both TSB and TSB containing 1 M NaCl to induce PGA production | |
