# Supplementary material for: Rapid Capsular Antigen Immunoassay for Diagnosis of Inhalational Anthrax: Preclinical Studies and Evaluation in a Nonhuman Primate Model
Source: mBio. 2022 May 12;13(3):e00931-22. doi: 10.1128/mbio.00931-22 (PMC9239138; doi:10.1128/mbio.00931-22)
Supplement: TABLE S4 [file mbio.00931-22-s0004.docx]

| **Supplementary Table 4. Evaluation of AAD with serum from patients with sepsis^a^** | | |
| --- | --- | --- |
| **Blood culture result** | **Number of patients** | **Positive AAD** |
| *Bacillus spp.* (not *anthracis*) | 4 | 0 |
| *Bacteroides thetaiotaomicron* | 1 | 0 |
| *Candida tropicalis* | 1 | 0 |
| *Enterococcus faecium* | 1 | 0 |
| *Escherichia coli* | 8 | 0 |
| *Klebsiella pneumoniae* | 2 | 0 |
| *Micrococcus luteus* | 1 | 0 |
| *Proteus mirabilis* | 2 | 0 |
| *Staphylococcus aureus* | 3 | 0 |
| *Staphylococcus auricularis* | 1 | 0 |
| *Staphylococcus capitis* | 3 | 0 |
| *Staphylococcus epidermidis* | 2 | 0 |
| *Staphylococcus haemolyticus* | 2 | 0 |
| *Staphylococcus hominis* | 7 | 0 |
| *Staphylococcus saprophyticus* | 1 | 0 |
| *Streptococcus gallolyticus* | 1 | 0 |
| *Streptococcus pneumoniae* | 2 | 0 |
| *Streptococcus viridans* | 2 | 0 |
| *Stenotrophomonas maltophilia* | 1 | 0 |
| ^a^ Sera from patients with culture-proven sepsis were purchased from BioIVT. | | |
